# Targeted capture enrichment assay for non-invasive prenatal testing of large and small size sub-chromosomal deletions and duplications

**DOI:** 10.1371/journal.pone.0171319

**Published:** 2017-02-03

**Authors:** Maria C. Neofytou, Kyriakos Tsangaras, Elena Kypri, Charalambos Loizides, Marios Ioannides, Achilleas Achilleos, Petros Mina, Anna Keravnou, Carolina Sismani, George Koumbaris, Philippos C. Patsalis

**Affiliations:** 1 Translational Genetics Team, The Cyprus Institute of Neurology and Genetics, Nicosia, Cyprus; 2 NIPD Genetics Ltd, Nicosia, Cyprus; 3 Department of Cytogenetics and Genomics, The Cyprus Institute of Neurology and Genetics, Nicosia, Cyprus; Tel Aviv University, ISRAEL

## Abstract

Noninvasive prenatal testing (NIPT) using whole genome and targeted sequencing has become increasingly accepted for clinical detection of Trisomy 21 and sex chromosome aneuploidies. Few studies have shown that sub-chromosomal deletions or duplications associated with genetic syndromes can also be detected in the fetus noninvasively. There are still limitations on these methodologies such as the detection of variants of unknown clinical significance, high number of false positives, and difficulties to detect small aberrations. We utilized a recently developed targeted sequencing approach for the development of a NIPT assay, for large and small size deletions/duplications, which overcomes these existing limitations. Artificial pregnancies with microdeletion/microduplication syndromes were created by spiking DNA from affected samples into cell free DNA (cfDNA) from non-pregnant samples. Unaffected spiked samples and normal pregnancies were used as controls. Target Capture Sequences (TACS) for seven syndromes were designed and utilized for targeted capture enrichment followed by sequencing. Data was analyzed using a statistical pipeline to identify deletions or duplications on targeted regions. Following the assay development a proof of concept study using 33 normal pregnancies, 21 artificial affected and 17 artificial unaffected pregnancies was carried out to test the sensitivity and specificity of the assay. All 21 abnormal spiked-in samples were correctly classified as subchromosomal aneuploidies while the 33 normal pregnancies or 17 normal spiked-in samples resulted in a false positive result. We have developed an NIPT assay for the detection of sub-chromosomal deletions and duplications using the targeted capture enrichment technology. This assay demonstrates high accuracy, high read depth of the genomic region of interest, and can identify deletions/duplications as small as 0.5 Mb. NIPT of fetal microdeletion/microduplication syndromes can be of enormous benefit in the management of pregnancies at risk both for prospective parents and health care providers.

## Introduction

Large and small size sub-chromosomal deletions and duplications are associated with genetic disorders and syndromes [1]. This group of clinically recognizable disorders is characterized by diverse phenotypes including intellectual disability (ID), autism and other neurodevelopmental disorders (NDD) [2]. Chromosomal abnormalities can result from genomic structural changes, such as copy number changes, leading to abnormal gene dosage with a dramatic impact on gene expression and phenotype [1].

Currently prenatal diagnosis of large sub-chromosomal deletions and duplications relies on invasive testing of fetal genetic material through chorionic villus sampling (CVS) or amniocentesis using karyotyping as the preferred method of analysis [3]. The evolution of whole genome microarray technologies has also enabled the detection of smaller pathogenic genomic rearrangements, which cannot be detected by conventional karyotyping [4–6]. However, invasive prenatal testing entails a modest, but significant risk of miscarriage of about 0.1–0.2% [7]. Prenatal screening, can now identify about 95% of pregnancies with Trisomy 21 [8]. Despite the fact that detection rates using the first-trimester combined test are relatively high for chromosomal aneuploidies, microdeletion and microduplication syndromes cannot be easily detected early in pregnancy [9]. Only certain ultrasound findings, such as large nuchal translucency (NT), and fetal cardiac defects are associated with increased risk of such conditions [10, 11]. The majority of clinically relevant deletions and duplications occur *de novo* and their combined incidence is high in the general population [6]. The most common microdeletion is 22q11.2 deletion syndrome also known as DiGeorge syndrome, with an incidence of 1 in 992 pregnancies in the low-risk population [12]. This incidence rate is higher than both Edwards (trisomy 18) and Patau (trisomy 13) syndromes’ [13]. Noninvasive prenatal screening and early detection of these sub-chromosomal imbalances is important, as it will help identify high risk pregnancies and offer the possibility of a confirmatory invasive diagnostic test after counseling. Such knowledge can offer better clinical management during pregnancy and after birth, where early intervention can potentially improve the quality of life of the newborn [14, 15].

With the recent discovery of cell-free fetal DNA (cffDNA) in maternal circulation, new possibilities of noninvasive prenatal testing (NIPT) became available. In an important study, Lun et al., showed that during the first trimester of pregnancy approximately 10% cffDNA is present in maternal plasma [16]. Sequencing of cffDNA can be used to identify the genetic and mutational profile of the fetus [17]. Cell free DNA (cfDNA) testing using whole genome, and targeted sequencing technologies [18, 19] and epigenetic based approaches [20, 21] has become increasingly accepted for routine clinical detection of Trisomy 21, single gene disorders, and X-linked conditions [22–26]. Since 2011, the American College of Obstetricians and Gynecologists and the Society for Maternal-Fetal Medicine recommends cfDNA testing as a highly accurate screening option for women at increased risk of fetal aneuploidy [27].

Several groups utilized whole genome sequencing of maternal plasma DNA and showed the high potential of this technology to detect fetal microdeletion syndromes [28–30]. Translocations and microduplications have also been detected across the fetal genome using next generation sequencing (NGS) [31]. More recently, a study published by Zhao et al., using low-coverage whole genome sequencing reported the detection of deletions ranging from 3 to 40Mb [32]. Undoubtedly whole genome sequencing based NIPT methodologies can reveal deletions or duplications with unknown clinical significance and a high number of false positive results, mainly due to the very low read depth of whole genome sequencing and the non-targeted nature of the test [33]. Furthermore, targeted sequencing based NIPT approaches have used SNPs for the detection of large deletions that underlie common syndromes [34]. A recent clinical evaluation study of the proposed SNP-based NIPT assay for 22q11.2 deletion syndrome reported high false positive rates suggesting the need of a reflex test (i.e. deep sequencing) in high-risk samples [35].

There is great need for the development of an accurate non-invasive prenatal test that can identify sub-chromosomal deletions and duplications. In this study we have developed and assessed a highly accurate and cost effective NIPT assay for the detection of sub-chromosomal small and large size deletions and duplications using targeted capture enrichment technology. This assay exhibits very high read depth in genomic regions of interest and allows the robust and accurate detection of sub-chromosomal aberrations as small as 0.5Mb in size.

## Materials and methods

### Ethics statement

The study has been approved by the Cyprus National Bioethics Committee. All samples were collected anonymously following written informed consent.

### Sample collection and cfDNA extraction

Peripheral venous blood (8mL), in EDTA-containing tubes, from women with singleton euploid pregnancies and samples from non-pregnant women were obtained from the Translation Genetics Team biobank of the Cyprus Institute of Neurology and Genetics. Plasma samples were obtained from pregnancies between 11^th^-14^th^ weeks of gestation, carrying fetuses of either gender. Within 4 hours after venipuncture, blood samples were subjected to centrifugation at 1,600 X g for 10 min at 4°C. The separated plasma portion was obtained and was re-centrifuged at 16,000 X g for 10 min at room temperature. Following this, plasma samples were stored at -80°C until further processing. Circulating cfDNA was extracted from 4 mL plasma using the QIAsymphony SP instrument and the QIAsymphony DSP Virus/Pathogen Midi Kit (Qiagen, Hilden, Germany) following the manufacturer’s instructions (QIAsymphony DSP Virus/Pathogen Midi Kit Handbook). Affected samples were generated using samples from individuals diagnosed with Wolf-Hirschhorn, and Potocki-Lupski syndrome (17p11.2 duplication syndrome) that were obtained from the Coriell Cell Repositories (Camden, NJ). Affected spiked-in samples were also generated for 1p36 deletion, Smith-Magenis, Miller-Dieker (MDS) 22q11.2 deletion syndrome and NF1 microdeletion syndromes using DNA samples acquired from the Cyprus Institute of Neurology and Genetics biobank (Table 1).

**Table 1:** Affected samples used to create artificial plasma pregnancies with deletion or duplication syndromes.

Sample ID	Location Identified	Method	Disorder	Type	Size (Mb)	OMIM (#)
NA23053	arr17p13.1p11.1(11096921–22159777)x3	aCGH	Potocki-Lupski	Dup	11.06	610883
NA22601	arr4p16.3p15.2(55665–25591051)x1	aCGH	Wolf-Hirschhorn	Del	25.53	194190
C100	arr1p36.33p36.22(554298–11122093)x1	aCGH	1p36 deletion	Del	10.56	607872
C101	arr17p13.3(48569–2002395)x1	aCGH	Miller-Dieker	Del	1.95	247200
C102	arr22q11.21(18706023–21505380)x1	aCGH	22q11.2 deletion syndrome	Del	2.79	188400
C103	arr17p11.2(16704279–20270654)x1	aCGH	Smith-Magenis	Del	3.56	182290
C104	arr17q11.2(28999864–30374607)x1	aCGH	NF1 microdeletion	Del	1.37	613675

### Preparation of artificial affected/unaffected plasma samples and fetal fraction estimation

Due to the low prevalence of detected pregnancies with the syndromes of interest, affected plasma samples were constructed *in vitro*. DNA from the affected individual, with known deletion or duplication syndrome, was spiked in isolated plasma DNA of non-pregnant women at concentrations of 20%, 10%, and 5%. Unaffected samples were generated following the same principle using normal genomic DNA. Before mixing, affected genomic DNA was sheared using sonication (Diagenode) at an average size of 200bp to simulate cell free fetal DNA in maternal plasma [36].

The concentration of spiked-in DNA into plasma cfDNA was measured following the preparation of the samples. Real-time PCR was performed using Taqman probes targeting the DYS14 and beta-globin loci as previously described [37]. A statistical model that utilizes allelic counts at heterozygous loci in maternal plasma was used for fetal fraction estimation in all 33 normal pregnancy samples in the proof of concept study [23].

### Library preparation

We used DNA from artificially affected pregnancies and actual pregnancies to generate libraries for the Illumina NGS platform as described in Mayer and Kircher [38]. Each library was barcoded using unique adaptor sequences to allow subsequent discrimination after sequencing. Negative controls were used to monitor potential contamination introduced during the preparation of samples. Barcodes were added to the samples using Herculase II Fusion Polymerase (Agilent Technologies) and were purified using the Qiaquick PCR Purification Kit (Qiagen, Hilden, Germany) [39].

### Design and construction of Target Capture Sequences (TACS)

Target Capture Sequences (TACS) were used to enrich regions of interest from the already prepared Illumina libraries using in solution hybridization. Primers were designed to amplify unique genomic loci that span the critical region of each syndrome as defined by the DECIPHER Database [40]. Additional primers were designed to amplify unique regions on autosomal chromosomes to be used as a reference for sub-chromosomal deletion/duplication detection (Table 2). Primers for each target region were designed using Geneious 6.1.8, [41] to yield amplicon sizes of 250bp with similar GC% content. Genomic regions that include repetitive elements were excluded. For the preparation of TACS, polymerase chain reaction was performed using MyTag polymerase (Bioline, London, UK) followed by purification, as previously described [23]. TACS were quantified using NanoDrop-ND8000 (Thermo Scientific, Wilmington, DE, USA) and were pooled equimolarly. The final mix was blunt-ended using the Quick Blunting kit (New England Biolabs) and was biotinylated by ligating a Bio-T/B adapter. Biotinylated TACS were purified using the MinElute kit (Qiagen) and were immobilized on streptavidin-coated magnetic beads (Invitrogen) as described in Maricic et al., [42].

**Table 2:** Number of TACS designed on critical microdeletion/microduplication regions and on reference chromosomes.

Syndrome/Overlapping Syndrome	Chr.	Location (GRch37)	Critical Region Size* (Mb)	No. TACS
1p36 microdeletion Syndrome	1	10001–12840259	12.83	176
Wolf-Hirschhorn Syndrome	4	1569197–2110236	0.54	70
Miller-Dieker Syndrome	17	1–2588909	2.59	138
Smith-Magenis Syndrome/ Potocki-Lupski Syndrome	17	16773072–20222149	3.45	132
NF1 microdeletion Syndrome	17	29107097–30263321	1.4	99
22q11.2 deletion Syndrome (Velocardiofacial, DiGeorge syndrome)	22	19009792–21452445	2.44	140
Total Number of TACS on syndromic regions				755
Total Number of TACS used as Reference Chr1-Chr12				490

* according to DECIPHER Database.

### In solution hybridization enrichment

Each library was mixed with Agilent 2X hybridization buffer (Agilent Santa Clara, CA), Agilent 10X blocking agent (Agilent), blocking oligonucleotides, Cot-1 DNA (Invitrogen) and salmon sperm DNA (Invitrogen) [23, 42]. The mix was heated to 95°C for 3 min to separate the DNA strands and was incubated at 37°C for 30 min before addition to the biotinylated TACS. The mixture was then incubated at 65°C for 48h. After hybridization, unbound DNA was washed away and captured sequences were eluted by heating as previously described [39]. Enriched sequences were amplified using outer-bound primers and samples were sequenced on a HiSeq 2500 sequencing platform (Illumina, San Diego, USA).

### Bioinformatics analysis

Sequencing reads were aligned to the human genome reference sequence (GRCh37/hg19) using the BWA-MEM aligner [43]. Initially, we used cutadapt to remove adapter sequences and performed quality control to the sequences before the alignment using FastQC [44]. Post alignment, Picard was used to sort the resulting BAM files and remove duplicate reads. Local realignment and base recalibration was performed using GATK and the read depth of each target region was retrieved using SAMtools [45, 46].

### Statistical testing for classification

Testing for microdeletions/microduplications is equivalent to testing for statistically significant differences between the read depth of the TACS in tested region (i.e. the part of the critical region covered by the TACS) and the read depth of the reference TACS. In the assay development part of our study we evaluated the importance of having an increased number of TACS in the reference region. To this end, we considered two methods that are essentially different in the number of TACS used in the reference region. In the first method, the reference region comprised of the TACS designed on the critical regions other than the tested region and on an additional 490 TACS designed on chromosomes 1–12. In the second method the 490 additional TACS are excluded thus leaving only 755 TACS (Table 2) in total (for both reference and test regions). Only the second method, which included 755 TACS, was used in the proof of concept study. Irrespective of the number of TACS used, a paired t-test was applied between the read depth of the TACS in the testing region and the median read depth of the TACS in the reference region. Due to the large number of TACS the corresponding scores from the test are asymptotically normal and thus can be considered as Z-scores. These scores were further normalized, both regarding location as well as scale. The former was achieved by removing the median score across all samples while the latter was achieved by dividing by the empirical standard deviation of each syndrome. The location normalization was applied on a sequencing run basis in order to additionally alleviate discrepancies between sequencing runs. The empirical standard deviation was estimated from the scores of the normal samples in our study. In more detail, let t_1_^k^,t_2_^k^,..,t_n(k)_^k^ denote the t-test statistics obtained by applying the paired t-test in the n(k) samples of run k. We defined run median, M^k^, as the median value of t_1_^k^,t_2_^k^,..,t_n(k)_^k^. The empirical standard deviation, SD_norm_, is calculated as the sample deviation of the t-test statistics that correspond to normal samples (i.e., without subchromosomal aneuploidies) across all runs. Location normalization refers to the subtraction of M^k^ from all t-test statistics of run k and scale normalization refers to the division of all t-test statistics, irrespective of run, by SD_norm_. Thus, the normalized z-score, z_i_^k^, is obtained using the formula z_i_^k^ = (t_i_^k^-M^k^)/SD_norm._

Raw scores of spiked samples and normal pregnancies are provided in the supporting information (S1 Appendix). The threshold was set to -3 for microdeletion testing and 3 for microduplication testing. After scale normalization, this corresponds to a threshold of 3 standard deviations.

## Results

### Optimization of common microdeletions and Potocki-Lupski syndrome detection using artificial pregnancies

To test the performance of the current targeted capture enrichment approach for detecting common microdeletions and the Potocki-Lupski syndrome in plasma, we generated artificially affected plasma samples for each microdeletion or microduplication syndrome. The assay development of the study was performed using 21 artificially affected plasma samples and 18 artificially unaffected plasma samples. All samples underwent enrichment using a pool of 490 TACS on reference chromosomes (chr1-chr12) and 755 TACS on syndromic regions. In total 37 out of 39 spiked samples were analyzed. Two samples were excluded from analysis due to low coverage (<10x) (Table 3). Analysis was performed using the two approaches, described in Statistical Testing for classification, to determine the optimal approach for accurate deletion/duplication detection. The results from the two methods are summarized in Table 3 and can be seen in the supporting information (S1 and S2 Figs).

**Table 3:** Assay development analysis 1 using 1245 TACS and analysis 2 using 755 TACS.

	Assay Development Analysis 1 (TACS = 1245)				Assay Development Analysis 2 (TACS = 755)			
Syndrome Tested	True Positives	False Positives	True Negatives	False Negatives	True Positives	False Positives	True Negatives	False Negatives
1p36 deletion	3	0	18	0	3	0	18	0
Wolf-Hirschhorn	2*	0	18	0	2	0	18	0
Miller-Dieker	3	0	18	0	3	0	18	0
Smith-Magenis	3	0	18	0	3	0	18	0
Potocki-Lupski	3	0	18	0	3	0	18	0
NF1-microdeletion	3	0	18	0	3	0	18	0
22q11.2 deletion syndrome	2*	0	18	0	2	0	18	0

* Two spiked samples (10% of Wolf-Hirschhorn and 10% of 22q11.2 deletion syndromes) were excluded from analysis due to low sequencing coverage.

In order to confirm the 20%, 10% and 5% simulated fetal fraction of each spiked sample, we performed qPCR as previously described [37, 47]. The concentration of the spiked-in DNA was estimated using Y-chromosome sequences. The observed fetal fraction ranged from 4% to 26%.

### Analytical proof of concept of fetal microdeletions and Potocki-Lupski syndrome detection

**Fig 1:**
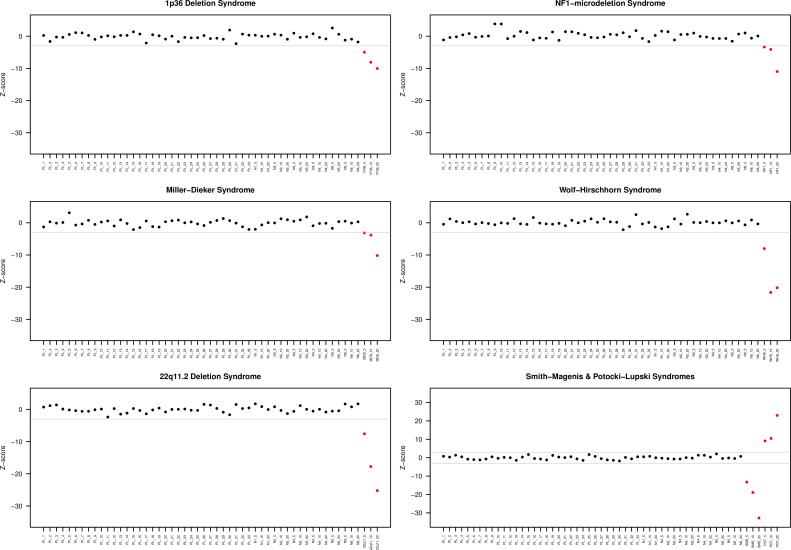
Detection of common microdeletions and Potocki-Lupksi syndrome using affected and unaffected spiked samples and normal pregnancy samples. Plots display the Z-scores used for status classification. In all plots, red dots indicate affected samples and black dots unaffected samples. The threshold (grey line) was set to 3 standard deviations after score normalization and was negative for microdeletions and positive for microduplications. In all syndromes, affected samples passed the threshold, while unaffected did not, resulting in 100% sensitivity and specificity. PL: normal maternal plasma N: normal spiked sample 1p36: 1p36 deletion spiked sample NF1: NF1 microdeletion spiked sample POT: Potocki-Lupski spiked sample SMS: Smith-Magenis spiked sample MDS: Miller-Dieker spiked sample WHS: Wolf-Hirschhorn spiked sample 22q11: 22q11.2 deletion syndrome spiked sample.

Following the initial design study for the development of the assay, a blind proof of concept study was performed to assess the sensitivity and specificity of the assay. A set of affected spiked samples, and plasma samples from normal pregnancies were used. Twenty one plasma samples spiked-in with DNA from microdeletions or the Potocki-Lupski duplication syndromes, 17 plasma samples spiked with normal DNA, and 33 normal pregnancy samples were tested using the Z-score based statistical analysis that included the 755 optimal TACS. Microdeletions or the selected duplication syndrome were clearly detected in all abnormal spiked samples without any false negative events (Fig 1) (Table 4). All 33 samples from normal pregnancies and the 17 plasma samples spiked with unaffected DNA were classified as normal for all syndromes (Fig 1). Fetal fraction estimation for the 33 normal pregnancy samples, ranged from 2% to 22.5% with mean fetal fraction of 11.6% (std.dev = 0.05). All fetal fraction estimates can be found in supplementary table (S1 Table).

**Table 4:** Detection rate and false positive rate for microdeletion and microduplication of artificially affected samples.

Syndrome Tested	True Positives	False Positives	True Negatives	False Negatives
1p36 deletion	3	0	50	0
Wolf-Hirschhorn	3	0	50	0
Miller-Dieker	3	0	50	0
Smith-Magenis	3	0	50	0
Potocki-Lupski	3	0	50	0
NF1- microdeletion	3	0	50	0
22q11.2 deletion syndrome	3	0	50	0

## Discussion

Non-invasive prenatal testing has focused mainly on whole chromosome aneuploidies and has advanced rapidly in clinical practice. Recent microarray studies showed that clinically relevant deletions or duplications are found in 1.7% of pregnancies with clinical indications for prenatal diagnosis [6]. Whole genome sequencing and targeted sequencing of cffDNA found in maternal circulation has enabled the detection of fetal sub-chromosomal events avoiding the risk of miscarriage [28, 29, 32].

However, there are still limitations when using the current methodologies to detect fetal specific sub-chromosomal deletions and duplications. A major limitation of all the current methodologies is their inability to detect small-size pathogenic aberrations [32, 33]. Whole genome approaches require high read depth to achieve statistical significance in their positive calls. This increases the cost substantially and renders such approaches inappropriate for clinical implementation [31]. Independent studies of the commercially available NIPT assays revealed a high number of false positive results, and ambiguous interpretation of positive results due to the identification of variants of unknown clinical significance [48]. Recently, a novel targeted capture enrichment assay has been developed and has been validated for the detection of fetal chromosomal aneuploidies [23]. In the present study, we demonstrated that this methodology could be adapted to accurately detect small and large size sub-chromosomal fetal deletions/duplications using maternal plasma.

A panel that includes 1p36 deletion, 22q11.2 deletion, Smith-Magenis, Potocki-Lupski, Miller-Dieker, NF1 microdeletion and Wolf-Hirschhorn syndromes was designed and tested. The study exhibited highly accurate detection of all 6 common microdeletions and Potocki-Lupski syndrome using affected and unaffected samples constructed artificially, as well as cfDNA samples from normal pregnancies. The assay is characterized by very high read depth, which results in highly accurate deletion/duplication detection. It avoids CNVs of unknown clinical significance and has the ability to identify deletions or duplications in the fetus smaller than 1Mb in size.

As previously mentioned, one of the main limitations of existing NIPT methods is the lack of sensitivity for detecting small size sub-chromosomal events [49]. Wapner et al., reported the detection of deletions associated with five common syndromes using informative SNPs, however all syndromes were caused by large deletions [34]. Current NIPT approaches report high accuracy for detecting deletions/duplications larger than 5Mb while detection rates fall dramatically for aberrations which are smaller than 5Mb [30, 33, 48]. The majority of deletions/duplications in most syndromes are smaller than 5Mb. According to published data, ~96% of affected individuals with 22q11.2 deletion syndrome have a defined 1.5-3Mb deletion [50]. To overcome one of the major testing limitations for microdeletion syndromes, we targeted small size deletions (Table 2). In the case of Wolf-Hirschhorn syndrome, the sample obtained had a 25Mb deletion, encompassing the syndrome’s 0.5Mb critical region. As such, TACS were designed to target specifically the 0.5Mb critical region, hence, for the purpose of this study this was the size of the microdeletion under investigation. Based on the results obtained, we have accurately detected the aforementioned aberration in all affected samples, therefore, it can be concluded that the assay allows the robust and accurate detection of chromosomal aberrations as small as 0.5Mb in size. The main factor that impacts the detection of sub-chromosomal imbalances in our assay is the size and the genomic architecture of the targeted region. TACS were designed to avoid GC rich regions that can have adverse effects during enrichment and to avoid repetitive elements that can consume large amounts of sequencing output [32, 51].

Using the same approach we have successfully detected a sub-chromosomal duplication associated with Potocki-Lupski syndrome in all spiked-in samples. To our knowledge this is the first study in which detection of microduplication syndrome using a targeted NGS approach is reported. According to DECIPHER there are 10 known duplication syndromes including the 22q11.2 duplication syndrome and the 1q21.1 recurrent microduplication syndrome associated with intellectual disability and dysmorphic features [40]. In order to expand the panel to enable the detection of other microduplications, additional TACS can be designed and incorporated in the pool of TACS.

Fetal fraction estimation in maternal plasma is a very critical parameter, which is associated with the accuracy of NIPT [52, 53]. We have incorporated in our NIPT assay a previously developed algorithm that accurately estimates fetal fraction in both male and female samples. The mean fetal fraction of all maternal plasma samples was 12% and is in agreement with previously published data from pregnancies at 11–13 weeks of gestation [54]. The use of accurate fetal fraction estimation enables the identification of pregnancies with low fetal fraction. These low fetal fraction samples should be excluded from NIPT analysis to minimize the possibility of false negative results [52]. In our cohort of artificially affected plasma samples, the underlying microdeletions and microduplications were identified in all samples. As expected, the classification power increases as fetal fraction increases, resulting in higher separation between normal and abnormal samples (Fig 1). We observed robust detection of microdeletion/microduplication in the 20%, 10%, and 5%ff samples, suggesting that affected fetuses can be identified as early as in the first trimester of gestation. The assay development and proof of concept of this methodology was performed using plasma samples from normal pregnancies and serial dilutions of affected and unaffected DNA due to the lack of maternal plasma samples from affected pregnancies. We recognize that a validation study is needed using a sufficient number of high-risk and affected maternal samples to accumulate more data and thoroughly assess the performance of our method.

Since pathogenic sub-chromosomal deletions and duplications occur *de-novo*, it is suggested to expand the use of cfDNA testing for microdeletion/microduplication syndromes from high-risk pregnancies to the general pregnancy population [15]. This would require careful and thorough counseling before testing, and a comprehensive evaluation of all ethical and socioeconomic aspects. Parental counseling should include the frequency of each syndrome, the phenotypic consequences, and a thorough explanation of the wide phenotypic heterogeneity of each condition, as well as the importance of careful diagnostic follow up [55]. It should be emphasized that women with a high risk result after NIPT should undergo diagnostic prenatal and postnatal testing with high resolution methods to determine the origin and precise size of the genomic alteration.

## Conclusions

We have developed and assessed an accurate and cost-effective assay for the NIPT of sub-chromosomal small and large size deletions and duplications using targeted capture enrichment technology. The use of TACS on selected syndromic regions offers high multiplex capabilities reducing the sequencing cost per base for each sample. In addition this approach requires less time for bioinformatics analysis since it is limited to a specific number of genomic regions. This assay exhibited very high accuracy, resulting from the inherently very high read depth of the targeted microdeletion/microduplication genomic regions. Due to its targeted nature, the assay avoids the detection of CNVs of unknown clinical significance and enables the detection of deletions or duplications as small as 0.5Mb in size. The analytical performance of the assay was successfully evaluated using cfDNA samples from normal pregnancies and serial dilutions of abnormal DNA in plasma. These results indicate that detection of small and large size deletions and duplications is feasible. Additional validations with prospective and retrospective studies using affected plasma pregnancies should be performed to obtain diagnostic sensitivity and specificity rates. Early stage noninvasive identification of microdeletion and microduplication syndromes is clinically important as it empowers prospective parents to make informed decisions and enables health professionals to offer optimal pregnancy management, and postnatal interventions with long term improvements in the health of the newborn.

## Supporting information

S1 Appendix Raw Scores for all samples tested. (XLSX)

S1 Fig Assay Development Analysis 1 (1245 TACS). (TIFF)

S2 Fig Assay Development Analysis 2 (755 TACS). (TIFF)

S1 Table Fetal Fraction estimation. (DOCX)
