# Supplementary figures and images for: Targeted capture enrichment assay for non-invasive prenatal testing of large and small size sub-chromosomal deletions and duplications
Source: PLoS One. 2017 Feb 3;12(2):e0171319. doi: 10.1371/journal.pone.0171319 (PMC5291539; doi:10.1371/journal.pone.0171319)

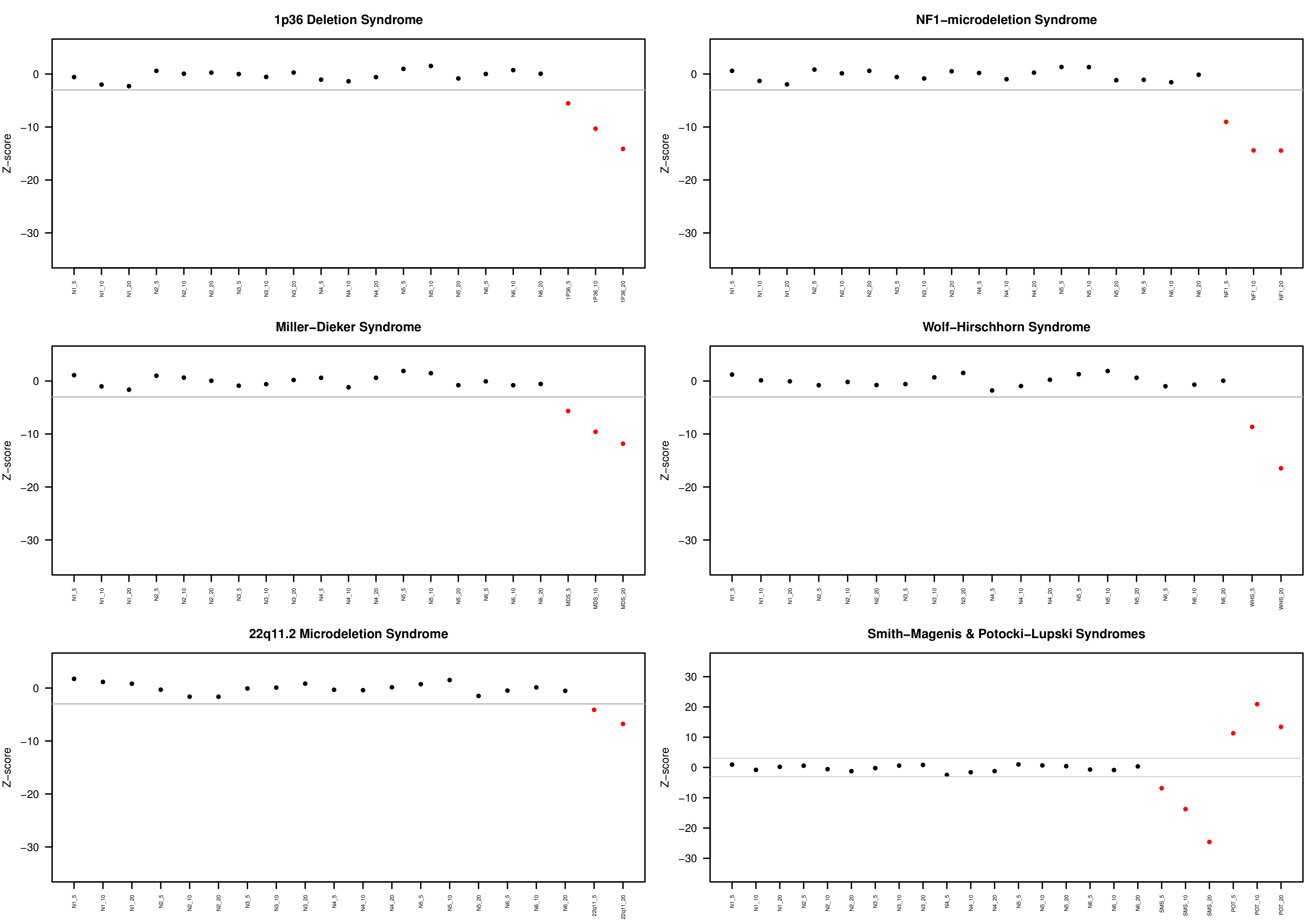

Supplement: S1 Fig — (TIFF) [file pone.0171319.s002.tiff]

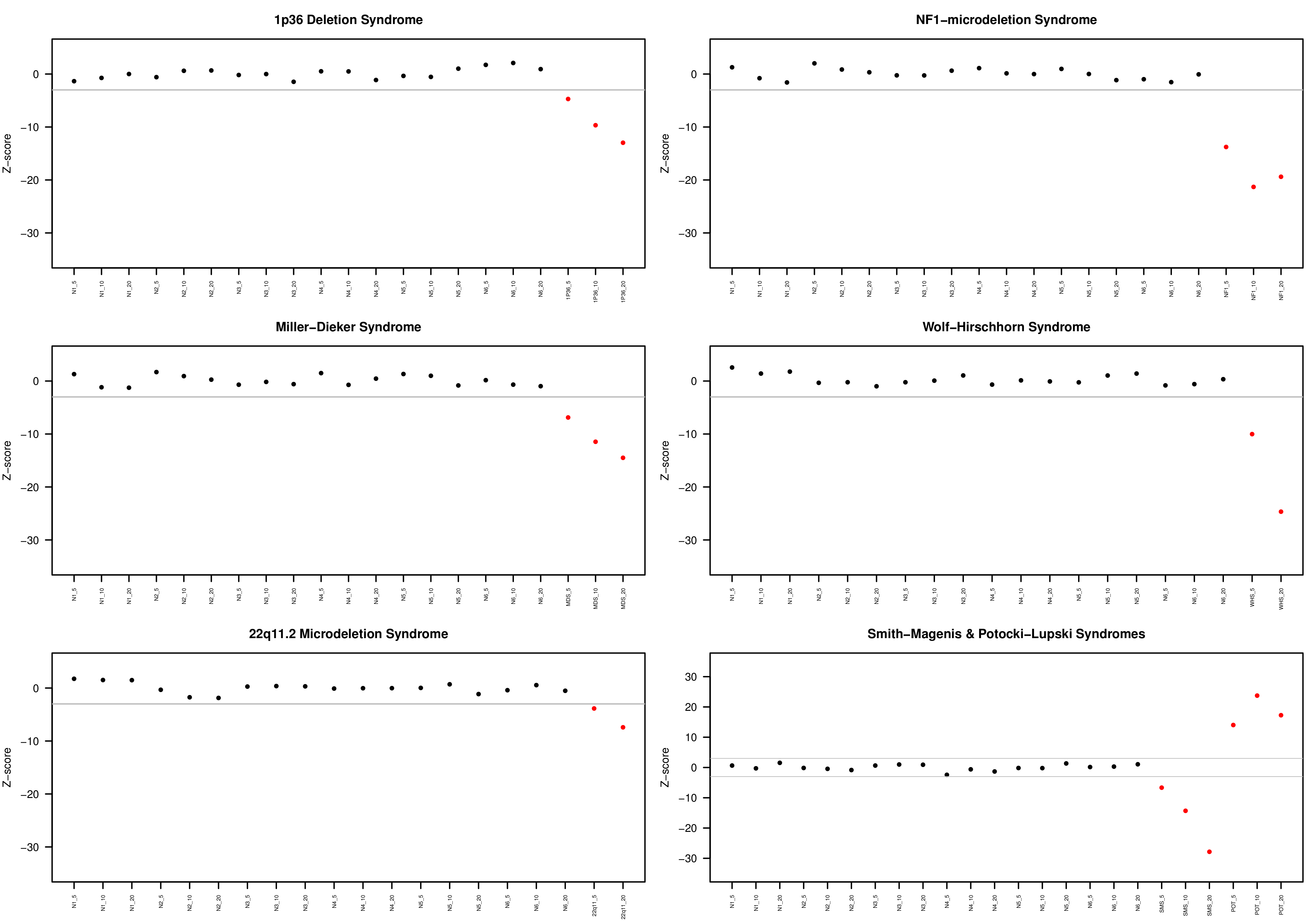

Supplement: S2 Fig — (TIFF) [file pone.0171319.s003.tiff]
