# Supplementary material for: Targeted capture enrichment assay for non-invasive prenatal testing of large and small size sub-chromosomal deletions and duplications
Source: PLoS One. 2017 Feb 3;12(2):e0171319. doi: 10.1371/journal.pone.0171319 (PMC5291539; doi:10.1371/journal.pone.0171319)
Supplement: S1 Table — (DOCX) [file pone.0171319.s004.docx]

**S1 Table. Fetal Fraction estimation using normal pregnancy samples and artificial affected and unaffected spiked samples.**

| **Sample ID** | **Fetal Fraction (%)** | **Method** |
| --- | --- | --- |
| Maternal plasma 1 | 13 | NGS |
| Maternal plasma 2 | 18 | NGS |
| Maternal plasma 3 | 10 | NGS |
| Maternal plasma 4 | 9 | NGS |
| Maternal plasma 5 | 14 | NGS |
| Maternal plasma 6 | 8 | NGS |
| Maternal plasma 7 | 11 | NGS |
| Maternal plasma 8 | 10 | NGS |
| Maternal plasma 9 | 10 | NGS |
| Maternal plasma 10 | 22 | NGS |
| Maternal plasma 11 | 7 | NGS |
| Maternal plasma 12 | 12 | NGS |
| Maternal plasma 13 | 11 | NGS |
| Maternal plasma 14 | 17 | NGS |
| Maternal plasma 15 | 6 | NGS |
| Maternal plasma 16 | 6 | NGS |
| Maternal plasma 17 | 9 | NGS |
| Maternal plasma 18 | 12 | NGS |
| Maternal plasma 19 | 8 | NGS |
| Maternal plasma 20 | 21 | NGS |
| Maternal plasma 21 | 8 | NGS |
| Maternal plasma 22 | 6 | NGS |
| Maternal plasma 23 | 9 | NGS |
| Maternal plasma 24 | 7 | NGS |
| Maternal plasma 25 | 8 | NGS |
| Maternal plasma 26 | 23 | NGS |
| Maternal plasma 27 | 10 | NGS |
| Maternal plasma 28 | 12 | NGS |
| Maternal plasma 29 | 19 | NGS |
| Maternal plasma 30 | 17 | NGS |
| Maternal plasma 31 | 2 | NGS |
| Maternal plasma 32 | 9 | NGS |
| Maternal plasma 33 | 22 | NGS |
| NF1 spiked 20% | 26 | qPCR |
| NF1 spiked 10% | 14 | qPCR |
| NF1 spiked 5% | 8 | qPCR |
| MDS spiked 20% | 26 | qPCR |
| MDS spiked 10% | 12 | qPCR |
| MDS spiked 5% | 6 | qPCR |
| WHS spiked 20% | 21 | qPCR |
| WHS spiked 10% | 10 | qPCR |
| WHS spiked 5% | 5 | qPCR |
| N1_20% | 21 | qPCR |
| N1_10% | 8 | qPCR |
| N1_5% | 5 | qPCR |
| N2_20% | 17 | qPCR |
| N2_10% | 8 | qPCR |
| N2_5% | 4 | qPCR |
| N3_ 20% | 24 | qPCR |
| N3_10% | 10 | qPCR |
| N3_5% | 5 | qPCR |

NF1: NF1 microdeletion spiked sample MDS: Miller-Dieker spiked sample WHS: Wolf-Hirschhorn spiked sample N: unaffected spiked sample
